# Supplementary material for: Patient Acceptability of Home Monitoring for Neovascular Age-Related Macular Degeneration Reactivation: A Qualitative Study
Source: Int J Environ Res Public Health. 2022 Oct 21;19(20):13714. doi: 10.3390/ijerph192013714 (PMC9603709; doi:10.3390/ijerph192013714)
Supplement: Supplementary file 1 [file ijerph-19-13714-s001.zip › ijerph-1916263-supplementary file S1.pdf]

Supplementary File S1. Semi-structured guide for patient participant interviews

**Interview schedule**
1. Could you tell me about your experiences of going to the eye clinic for your regular check-ups?
2. Could you tell me about your reasons for wanting to take part in this study and to try out the tests at home?
3. What do you think about the purpose of the tests and of monitoring your vision at home?
4. What are your thoughts on monitoring at home if it meant people might have hospital appointments less often?
5. Would you be comfortable having hospital appointments less often?
6. What do you think about the idea of just monitoring at home, without hospital appointments?
7. How did you find the iinformation and training session at the start of the study?
8. How do you feel about using technology and electronic devices like mobile phones, tablets or computers?
9. What was your experience of using technology before the study?
10. Could you tell me about your experience of using each test?
11. Did you do the tests in any particular order?
12. How did you find doing the tests once per week? Was it too often or would it be ok to test more often?
13. Did you find testing was easy to do as part of your normal routine?
14. Was there anything that stopped you from testing at home?
15. How long did it usually take to do the tests each week?
16. Did you find the tests easy to use?
17. How confident were you that you could do the tests yourself?
18. Do you think others would be able to do the tests easily?
19. How suitable was the electronic device you were given for doing the home tests?
20. Di you find the novelty of testing at home wore off during study.
21. Did you use any support or help to do the tests? Do you still use the same amount of support after testing for some time?
22. Did you see any benefits from doing the tests?
23. Was there anything enjoyable about doing the tests?
24. Did you have to change any part of your home environment to do the tests.
25. Did you prefer any of the tests to the others?
26. What are your thoughts about receiving feedback scores for one of the tests (the numbers test) and not the shape test?
27. Where there any disadvantages of doing the tests?
28. Did you experience any technical issues with the tests?
29. Did the tests work all the time as they were meant to?
30. What would you change about the tests?
31. What advice would you give people doing the tests?
32. Would you like to add anything else or ask me any questions?
