# Supplementary material for: Patient Acceptability of Home Monitoring for Neovascular Age-Related Macular Degeneration Reactivation: A Qualitative Study
Source: Int J Environ Res Public Health. 2022 Oct 21;19(20):13714. doi: 10.3390/ijerph192013714 (PMC9603709; doi:10.3390/ijerph192013714)
Supplement: Supplementary file 1 [file ijerph-19-13714-s001.zip › ijerph-1916263-supplementary file S2.pdf]

Supplementary File S2. Framework for coding of interview transcripts

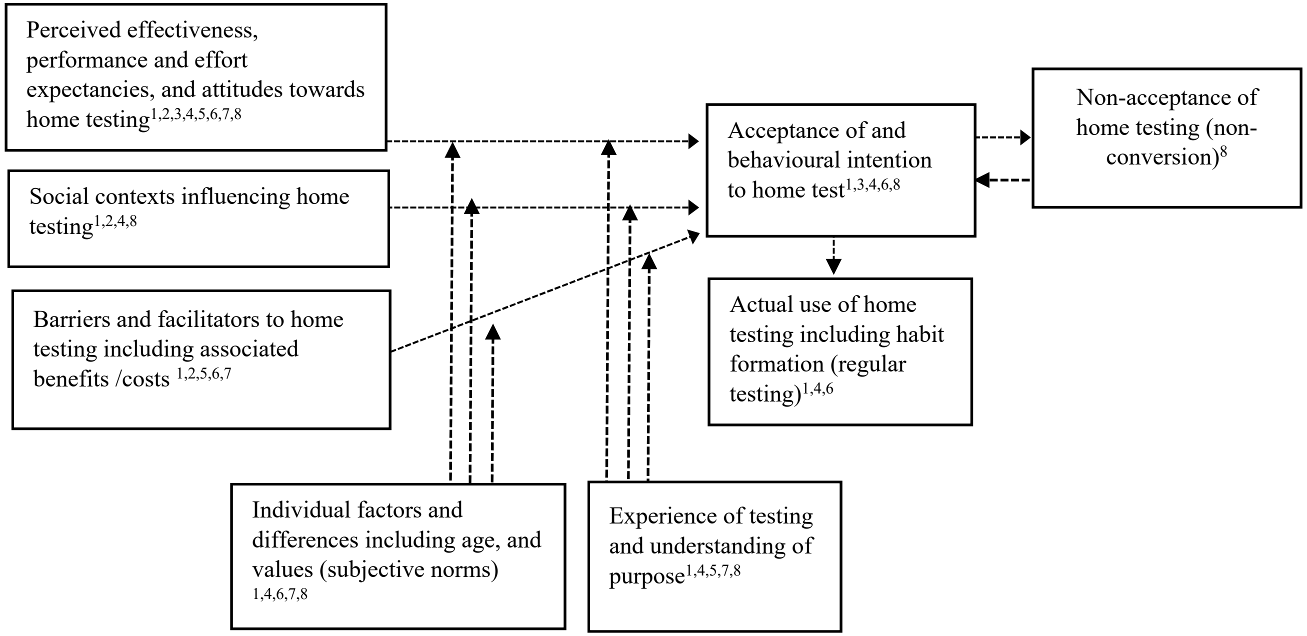

[1]Unified Theory of Acceptance and Use of Technology; [2]Extended Unified Theory of Acceptance and Use of Technology; [3]Technology Acceptance Model; [4]Technology Acceptance Model (TAM2); [5]Extended Technology Acceptance Model; [6]Extended Technology Acceptance Model for elderly people; [7]Theoretical Framework of Acceptability (v2)' [8]Senior Technology Acceptance and Adoption model (STAM)
