# Supplementary material for: Patient Acceptability of Home Monitoring for Neovascular Age-Related Macular Degeneration Reactivation: A Qualitative Study
Source: Int J Environ Res Public Health. 2022 Oct 21;19(20):13714. doi: 10.3390/ijerph192013714 (PMC9603709; doi:10.3390/ijerph192013714)
Supplement: Supplementary file 1 [file ijerph-19-13714-s001.zip › ijerph-1916263-supplementary file S3.pdf]

**Supplementary File S3. Final scheme for coding of interview data**

| | Code | Definition |
|---|---|---|
| 1 | Burden/Perceived amount of effort | The perceived amount of effort that is required to participate in home monitoring |
| 2 | Ethicality | The extent to which home monitoring is a good fit with an individual's value system |
| 3 | Intervention coherence | The extent to which the participant (mis)understands home monitoring and how it works |
| 4 | Opportunity costs | The extent to which benefits, profits or values must be given up to engage in home monitoring |
| 5 | Perceived effectiveness/perceived usefulness | The extent to which home monitoring is perceived as likely to achieve its purpose<br>An individual's perception that iPods (IT system) will enhance home monitoring (job performance) |
| 6 | Self-efficacy | The participant's confidence that they can perform the test(s) required; this also includes a lack of confidence |
| 7 | Perceived ease of use | An individual's perception that iPods to home monitor (IT system) is free of effort |
| 8 | Attitude | An individual's evaluative judgement of the target behaviour on some dimension (e.g. good/bad, harmful/beneficial, pleasant/unpleasant, also ambivalence) |
| 9 | Behavioural intention | An individual's motivation or willingness to exert effort to perform home monitoring |
| 10 | Actual behaviour/acceptance | The action of undertaking home monitoring |
| 11 | Subjective norm | An individual's perception of the degree to which important other people approve or disapprove of home monitoring |
| 12 | Facilitating or inhibiting conditions | Perception of availability of necessary skills, resources and opportunities for home monitoring. Also, organisational and technical infrastructure to support home monitoring. In context of MONARCH e.g. able to get a dark space for one app test which needs complete darkness, technical helpline etc. |
| 13 | Image | The degree to which an individual perceives that use of iPod home monitoring (an innovation) will enhance his or her status in his or her social system |
| 14 | Job relevance | The degree to which an individual believes that the home monitoring (target system) is applicable to his or her eye health (job) |
| 15 | Output quality | The degree to which an individual believes that the iPod (the system) performs his or her home monitoring (job tasks) well |
| 16 | Results demonstrability | The degree to which an individual believes that the results of iPod home monitoring (using a system) are tangible, observable, and communicable |
| 17 | Individual differences | Individual difference variables include personality and/or demographics (e.g., traits or states of individuals, gender, and age) that can influence individuals' perceptions of perceived usefulness and perceived ease of use. |

| | | |
|---|---|---|
| 18 | System characteristics | System characteristics are those salient features of iPod home monitoring (a system) that can help individuals develop favourable (or unfavourable) perceptions regarding the usefulness or ease of use of a system. |
| 19 | Technology apprehension | The degree to which an individual experiences apprehension, or fear, when she/he is faced with the possibility of using technology (computers) |
| 20 | Technology playfulness/comfortable with technology | The opposite of technology apprehension. The degree to which an individual is open to experiment, spontaneous interact or 'give technology a go' and comfort with technology |
| 21 | Technology persistence | Not at the same level as technology playfulness but participant appears willing to try to troubleshoot or rectify problems that may arise when using the iPod |
| 22 | Perceived enjoyment | The extent to which an individual perceives home monitoring to be enjoyable in its own right |
| 23 | Reported experience | Participant actual experience of rather than perception of effort required to test when using the iPod |
| 24 | Voluntariness | A choice rather than enforcement of iPod home monitoring |
| 25 | Technology reliability | Output quality in terms of data transfer and equipment reliability |
| 26 | General health status | Participant's perception of their general health status |
| 27 | Perceived threat | An individual's perception of severity and susceptibility that eye health may deteriorate e.g. AMD no longer treatable, will lose sight or progression of AMD to other eye |
| 28 | Health beliefs and concerns about eye health | An individual's beliefs and concerns around AMD |
| 29 | Habit | The reported experience and perception that home monitoring has/has not become habitual |
| 30 | Aging | Functional, health, sensory, cognitive and mobility changes associated with increasing age influencing home monitoring |
| 31 | Medical Services satisfaction | The extent to which participants are satisfied with current health care services for AMD |
| 32 | Affordability of health services | Refers to the affordability of health services e.g. private health care within NHS context |
| 33 | Comfort with health services | Refers to the psychological feelings of patients towards health services and hospital environment e.g. cleanliness of hospital |
| 34 | Professionalism of healthcare staff | Refers to knowledge, skills and interpersonal skills of healthcare staff |
| 35 | Safety of healthcare | Participant's perception of healthcare safety e.g. experienced medical teams, complete medical facilities, hospital security measures |
| 36 | Waiting time | Patient's perception of waiting time for appointments, treatment etc. |
| 37 | Information quality | Home monitoring can provide useful information |

| | | |
|---|---|---|
| 38 | Usual care versus home monitoring | The perception of an individual to choose the physical hospital environment over the 'virtual'/home monitoring hospital environment includes: recommending current hospital to others and continuing to use this hospital |
| 39 | Perceived security | Perceived risk or protection from the transfer, management and analysis of personal health information |
| 40 | Healthcare professional's (Doctor's) opinion (similar to Subjective norm) | Influence of healthcare professionals as they are perceived to be a point of expert authority |
| 41 | Motivational intention to participate in, or seek out latest research | Participant expresses an intention or gets involved in research |
| 42 | Views about training/preparation | Participant describes their experience and perception of the training session or preparing themselves for home monitoring |
| 43 | Other influencing factors | Participant makes references to factors not otherwise covered by codes within this framework |
| 44 | Family support (not Subjective norms) | Participant makes reference to the presence of family or a significant other |
| 45 | Emotion | Participant describes stress, anxiety or experience of trauma |
| 46 | Major life event | Participant refers to major life event |
| 47 | Social context | Participant describes their living space, residential area, whether or not they live with others. |
| 48 | Reported performance | Participant describes their performance on one or more tests |
| 49 | Keep Sight Journal | Participant describes features and perception of the paper-based KSJ |
| 50 | Multibit | Participant describes features and perception of the electronic 'numbers' Multibit test |
| 51 | My Vision Track | Participant describes features and perception of the electronic 'bumpy circles/odd one out' My Vision Track test |
| 52 | Check-in calls for non-testers | Participant describes having been contacted by CTEU due to non-regular testing |
| 53 | Experience with eye care | Participant describes what happens or what has happened when they have been in contact with their local Macular, optometry or ophthalmologist service |
| 54 | Order of test | Preferential or chronological order of tests |
| 55 | Travel | Participant describes how they get to their usual appointments, includes incurred expenses or parking difficulties |
