# Supplementary material for: Patient Acceptability of Home Monitoring for Neovascular Age-Related Macular Degeneration Reactivation: A Qualitative Study
Source: Int J Environ Res Public Health. 2022 Oct 21;19(20):13714. doi: 10.3390/ijerph192013714 (PMC9603709; doi:10.3390/ijerph192013714)
Supplement: Supplementary file 1 [file ijerph-19-13714-s001.zip › ijerph-1916263-supplementary file S4.pdf]

Supplementary File S4. Relationship between themes and coding of interview data

**Themes**	**The role of home monitoring**		**Suitability of procedures and instruments**	**Experience of procedures**			**Feasibility of regular home monitoring in usual service delivery**		**Impediments to home monitoring**	
Sub-themes	*Understanding purpose*	*Perceived impact on eye care*		*Training for home monitoring*	*Test preferences*	*Use of MBT test feedback and data*	*Frequency of home monitoring and habit formation*	*Use of ongoing support*	*Practical issues*	*Personal health and social factors*
Codes	Relevance to eye care	Perceived usefulness	Individual preferences and use of technology	Technology apprehension	Perceived ease of use	Perceived threat of deterioration	Burden/perceived effort	Check-in calls for non-testers	Technology reliability[3]	Health status[2]
	Threat of deterioration in vision	Job relevance	Technology apprehension	Information quality	Burden/ perceived effort	Results demonstrability	Technology persistence	Family support (not subjective norm)[2]	Burden/ perceived effort	Social context[2]
	Perceived usefulness	Waiting time a concern	Individual differences - Age [1]	Views on training	Actual behaviour/ acceptance	Job relevance	Habit		Output quality	Individual differences - Age [2]
	Intervention coherence	Comfort with services	Attitude		Self-efficacy	Reported performance	Self-efficacy			
		Professionalism of healthcare staff	Behavioural intention to HM		System characteristics	Self-efficacy	Actual behaviour/Acceptance			
		Reduced travel	Information quality		Reported experience		Family support (not subjective norm)[1]			
			Health status[1]		Technology reliability[1]					
			Image		Perceived enjoyment		Technology reliability [2]			
					Technology playfulness/ Comfort		Health professional opinion (similar to subjective norm)			

Numbers in brackets represent codes which were evident across different themes, and highlight the number of times the code emerged.
