# Supplementary material for: Patient Acceptability of Home Monitoring for Neovascular Age-Related Macular Degeneration Reactivation: A Qualitative Study
Source: Int J Environ Res Public Health. 2022 Oct 21;19(20):13714. doi: 10.3390/ijerph192013714 (PMC9603709; doi:10.3390/ijerph192013714)
Supplement: Supplementary file 1 [file ijerph-19-13714-s001.zip › ijerph-1916263-supplementary file S5.pdf]

Supplementary File S5. Perspectives of informal 'carers'

Views of informal 'carers'	Patient theme/sub-theme	Supporting quote(s) from informal carers
Home monitoring could be a way to reduce the frequency of clinic monitoring visits.	**Theme 1. The role of home monitoring** *Sub-theme 2: Perceived impact on eye care*	*'I would be in favour of this self-testing if it became a permanent thing, it would save the journeys back and forward to the hospital. The fact that (partner) is prepared to shut herself away for half an hour every Friday morning and do the test, it leaves me just as somebody to answer the phone or callers to the door, it saves us a lot of time and effort'* ('Carer' 10, Male)
Training was important but information recall deteriorated after home monitoring started. Partners should be involved in training so that they can support their husband/wife/partner if needed.	**Theme 3. Experience of home monitoring procedures** *Sub-theme 1: Training for home monitoring*	*'…so, he did need a bit of assistance and he thought he knew what he was doing after he tried it there (at the training session) but he'd forgotten a bit. If you had two people that were non-technically minded, that might be a bit of an issue, you know. if the person (a partner or carer) hadn't been to it, there would be less chance of them understanding how to help do the actual test'*. ('Carer' 05, Female)
Use of test feedback provided a way to monitor changes in vision. Changes in vision, including improvements in response to treatment may be reflected in test scores.	**Theme 3. Experience of home monitoring procedures** *Sub-theme 3: Use of test feedback and data*	*'…but we knew it was about time in to get another injection, know what I mean? I just found it interesting that she did seem to go downhill the two weeks before that appointment'*. ('Carer' 03, Male)<br><br>*'…other than the fact that maybe after the injection, he got higher scores'*. ('Carer' 01, Female)
A reduction in feedback scores could be due to deterioration in visual acuity. Patients applied their own thresholds to what was a meaningful change.	**Theme 3. Experience of home monitoring procedures** *Sub-theme 3: Use of test feedback and data*	*'…and she (partner) is a lot more confident with it (home monitoring) now but sometimes gets disturbed at the results because there was a period where the scores were good and consistent, which suggested that there was no deterioration. Then one week, she came down to me and said I don't know what went wrong but the results were terrible. Instead of being over 50% it dropped to 40%. I said, maybe you weren't feeling the best, let's leave it another week and the following week it was back up to 50%'*. ('Carer' 09, Male)

Support for home monitoring could be provided by partners or family members if they were more familiar with using technology. Experience of testing over time may reduce the need for this support.	**Theme 4. Feasibility of regular home monitoring in usual service delivery**<br>*Sub-theme 2: Use of ongoing support*	*'…there were times when messages came up on the screen but all it needed was a particular button to be pressed or whatever and she wasn't sure but I was able to sort it - it was simple little things like that. Eventually she found she had the ability and the confidence to do it. It would be very rare for me to have to help her now with the actual machine'.* ('Carer' 02, Male)<br><br>*'...we thought we could call in our grandchildren, if we have bother with how to work it? However, it seemed straightforward. I would be more used to technology than my husband; he would prefer to be in a book than on an iPod'.* ('Carer' 01, Female)
