# Supplementary material for: Patient Acceptability of Home Monitoring for Neovascular Age-Related Macular Degeneration Reactivation: A Qualitative Study
Source: Int J Environ Res Public Health. 2022 Oct 21;19(20):13714. doi: 10.3390/ijerph192013714 (PMC9603709; doi:10.3390/ijerph192013714)
Supplement: Supplementary file 1 [file ijerph-19-13714-s001.zip › ijerph-1916263-supplementary file S6.pdf]

Supplementary File S6. Perspectives of Healthcare Professionals

Healthcare Professional (HCP) views	Patient theme / sub-theme	Supporting quote(s) from HCPs
Factors such as age, and lack of familiarity with technology were linked to participant's initial interest in HM and engagement with testing.	**Theme 2. Suitability of procedures and instruments**	*'…I know how old they are before I'm approaching them and so there are misconceptions on my side because I see someone who's 80 and I think they may not be willing to participate, but they can be compared to somebody who's in their 60s or early 70s. But yes, I think older people, are just not used to an iPod'.* (HCP03, Female)<br><br>*'…I think they split into two groups. The first is definitely the elderly, this technology is beyond them and they almost take out their phones to show you what they are accustomed to. The others are keen and interested; they like the concept'.* (HCP06, Female)
Training was essential to success and to long-term engagement. It was recognised that training was information heavy and often had to be tailored based on previous experience with technology. Participants may require further training, not just a single session.	**Theme 3. Experience of home monitoring procedures**<br>*Sub-theme 1: Training for home monitoring*	*'…others are at a stage where they learn how to use technology and every piece of information is crucial. We spend a lot more time on those who are brand new users of technology because if we want a good starting point we will have more difficulties later if they are not able to remember'.* (HCP02, Female)<br><br>*'I think just another check in is needed, I don't think maybe the one visit is enough actually, maybe two are needed'.* (HCP05, Female)
Participants preferred the MultiBit test (which provides performance feedback**) and most found this to be positive (allowing self-monitoring) but there were also negatives as uncertainty over meaning of performance scores could lead to anxiety.	**Theme 3. Experience of home monitoring procedures**<br><br>*Sub-theme 3: Use of test feedback and data*	*'..for one of the patients that have withdrawn, one of the biggest things has been the MultiBit test, I would say, and the percentage score given at the end. I think a lot of people find that distressing because they perceive the number to be lower than what they would like, although what benchmark they are using I don't know'.* (HCP05, Female).<br><br>*'…that's something that nearly every single participant that you speak to says is something that really distresses them (how percent scores are calculated) but nobody really knew how it was derived. To tell patients not to pay too much heed to it because it's not necessarily derived from their visual performance was really useful'.* (HCP01, Female)

Home monitoring was seen as an acceptable part of patient care but may be resource intensive due to the technical support needed to support patients.	**Theme 4. Feasibility of regular home monitoring in usual service delivery** *Sub-theme 2: Use of ongoing support*	*'...because it is indeed involving a lot of resources, it will be probably less demanding if the patient will need to come to see the specialist every couple of months. But equally, we will need to have enough provision of support for them when it's needed and it should be straight forward access'* (HCP02, Female)<br><br>*'..we have an understanding that a patient may experience this (connection problems) at home and despite advising them over the phone, there's not always a straight-forward solution to it'.* (HCP08, Male).
Participants required additional support, through this was usually informal (delivered at follow-up visits or over the phone).	**Theme 4. Feasibility of regular home monitoring in usual service delivery** *Sub-theme 2: Use of ongoing support*	*'…we have to compensate when we speak with people on the phone but if we go to clinic we sometimes ask people to bring the iPod so we can offer guidance on the spot, in case it's something very simple to address'.* (HCP01, Female).
A mechanism for providing 'feedback*' messages (on test completion) would be beneficial (ensuring participants knew test data had transferred).	**Theme 5. Impediments to home monitoring** *Sub-theme 1: Practical issues*	*'…another thing fed back at every single follow up, pretty much without fail, is that they get no feedback in terms of whether their results have been received by the study team or not'.* (HCP03, Female).<br><br>*'…it doesn't matter how much you reassure that they would be contacted if results weren't being received. They is no substitute for even just a 'Thank you for completing the tests, your results have been sent' or something like that'.* (HCP01, Female).

* Healthcare Professionals were research ophthalmologists and nurses

** 'Feedback' refers to information on successful test completion.
