# Supplementary material for: Patient Acceptability of Home Monitoring for Neovascular Age-Related Macular Degeneration Reactivation: A Qualitative Study
Source: Int J Environ Res Public Health. 2022 Oct 21;19(20):13714. doi: 10.3390/ijerph192013714 (PMC9603709; doi:10.3390/ijerph192013714)
Supplement: Supplementary file 1 [file ijerph-19-13714-s001.zip › ijerph-1916263-supplementary file S7.pdf]

Supplementary File S7. Common issues reported by patients during home monitoring

Reported issues	Patient's responses to issues	Relevant constructs of technology acceptance theories	Definition of construct
Successful methods of finding a dark space when completing the MBT inhibited actual performance of the test.	Test at night-time or find/create a home area with no light (e.g. under stairs, placing a blanket or material overhead).	Opportunity Costs[a]<br>Burden[a]<br>Effort Expectancy[b]	Extent to which HM causes benefits or values to be given up<br>Perceived effort required to participate in HM<br>Extent to which HM is seen as easy to do
The need to charge the iPod device before every occasion of testing.	Devices left on charge or paper-based KeepSight journal completed before App-based tests.	Burden[a]<br>Effort Expectancy[b]<br>Output Quality[c]<br>Perceived Effectiveness[a]	Perceived effort required to participate in HM<br>Extent to which HM is seen as easy to do<br>Extent to which HM performs its task well<br>Extent to which HM is likely to achieve its purpose
Mobile internet connection for transferring test data appeared to function inconsistently and participants were not aware or were uncertain whether or not test data was received by the study.	Home broadband connections used as an alternative where feasible.	Affective Attitude[a]<br>Perceived Effectiveness[a]<br>Perceived Risk[b]<br>Output Quality[c]	How individuals feel about HM<br>Extent to which HM is likely to achieve its purpose<br>Perceived negative consequences associated with HM<br>Extent to which HM performs its task well
Other technical issues e.g. Apps resting during tests, software updates, and uncertainty among participants about how to undertake tasks such as updating software.	Use of study helpline for support or support from family members or friends	Self-efficacy[a]<br>Effort Expectancy[b]<br>Perceived Risk[b]<br>Output Quality[c]	Confidence in performing HM procedures<br>Extent to which HM is seen as easy to do<br>Perceived negative consequences associated with HM<br>Extent to which HM performs its task well
Difficult to establish a testing routine due to commitments including caring roles and work responsibilities.	Use of reminders and prompts including diaries, self-administered phone reminders and testing at a set time/day	Burden[a]<br>Ethicality[a]<br>Opportunity Costs[a]<br>Social Influence[b]<br>subjective norm[c]<br>Individual Differences[b]<br>Habit[b]	Perceived effort required to participate in HM<br>Extent to which HM has a good fit with the individuals' values<br>Extent to which HM causes benefits or values to be given up<br>Extent to which important others believe HM should be performed Extent to which individual factors influence HM<br>Intention to HM and continued use of HM
Feedback from tests led to anxiety if scores declined consistently and	Consider contacting optometry/ ophthalmology services.	Self-efficacy[a]<br>Intervention Coherence[a]	Confidence in performing HM procedures<br>Extent to which HM purpose and how it works is understood

were attributed to deterioration in vision.		Perceived Effectiveness[a]<br>Performance Expectancy[b]/<br>Job Relevance[c]<br>Results Demonstability[c]	Extent to which HM is likely to achieve its purpose<br>Extent to which HM will help with eye care<br>Extent to which result are observable and communicable
Problems or issues with testing - if they were not easy to overcome without support, could lead to testing becoming irregular or stopping.	Use of 'problem solving' and support from study helpline or family members or friends.	Self-efficacy[a]<br>Affective Attitude[a]<br>Burden[a]<br>Facilitating conditions[b]	Confidence in performing HM procedures<br>How individuals feel about HM<br>Effort required to participate in HM<br>Extent to which infrastructure exists to support HM
Eye patches that were used when undertaking testing caused discomfort and were a distraction, thereby affecting test performance.	Modifications made to the eye patch.	Burden[a]<br>Perceived Effectiveness[a]	Perceived effort required to participate in HM<br>Extent to which HM is likely to achieve its purpose

a Theoretical Framework of Acceptability; b Unified Theory of Acceptance and Use of Technology; c Technology Acceptance Model; HM: Home Monitoring.
